# Retiform hemangioendothelioma: a case series and review of the literature

**DOI:** 10.1186/s13256-021-02671-2

**Published:** 2021-02-17

**Authors:** Qurratulain Chundriger, Muhammad Usman Tariq, Shabina Rahim, Jamshid Abdul-Ghafar, Nasir Ud Din

**Affiliations:** 1 Department of Pathology and Laboratory Medicine, Aga Khan University Hospital, Karachi, Pakistan; 2 Department of Pathology and Clinical Laboratory, French Medical Institute for Mothers and Children (FMIC), Kabul, Afghanistan

**Keywords:** Retiform, Hemangioendothelioma, Dabska tumor, Angiosarcoma, Hobnail

## Abstract

**Background:**

Retiform Hemangioendothelioma (RH) is an extremely rare vascular tumor of intermediate biological behavior, which is prone to local recurrence but rarely shows metastasis to distant sites. It may harbor areas resembling Dabska tumor in some cases and angiosarcoma, which in its well differentiated form may exhibit similar pathological appearance in some areas, making it problematic to rule out a possibility of a malignant diagnosis on a core biopsy. Therefore, complete surgical resection with negative margins is essential for accurate diagnosis and local control.

**Results:**

In our series, two of the three Pakistani cases were in females, with an age range between 18 and 50 years. Our first patient presented with symptoms of cardiac compromise and pulmonary hypertension. Her computed tomography scan showed multiple tumor masses within the mediastinum. The second patient presented with an ulcerated lesion on his scalp, at right temple. The third patient presented with a hard growth on her left 4th toe which was amputated. Histologically, all cases exhibited retiform arborizing vascular spaces lined by bland endothelial cells with hobnail nuclei, characteristic of retiform hemangioendothelioma. Immunohistochemical markers CD31, CD34 and ERG confirmed the vascular nature of the tumor. The first and the second patient are alive and healthy at 4 and 7 months follow up respectively, while the third patient is lost to follow up.

**Conclusion:**

Owing to the rate of local recurrence, RH should always be considered in the differential diagnosis of vascular tumors showing arborizing blood vessels, as it may have an atypical presentation and it should be carefully differentiated from Dabska tumor and an angiosarcoma.

## Background

According to the 5th edition of World Health Organization (WHO) classification of tumors of soft tissue and bone, vascular tumors are classified as benign, intermediate and malignant. The intermediate category is further divided into those with locally aggressive behavior and those which rarely metastasize. Retiform Hemangioendothelioma (RH) is a rare vascular neoplasm which belongs to the rarely metastasizing subgroup of intermediate category [1]. It commonly involves the skin and subcutaneous tissue of the lower extremities in patients over a wide age range. However, children and young adults are commonly affected. Uncommon sites of involvement include head and neck region, trunk, penis and pleura [2, 3]. Microscopically, the tumor is composed of arborizing vascular spaces which resemble the normal rete testis on low power, as the name implies. The vascular spaces are lined by single layer of endothelial cells with prominent hobnail nuclei projecting into the lumen. Most cases show local recurrence. Distant metastasis is extremely rare. Two of the earliest cases have reported to have metastasized to lymph nodes [2, 4]; while, metastasis to liver has only been reported in a single case [5]. We herein describe the clinicopathological and histological features of 3 cases of RH with thorough review of literature which will help us in establishing correct diagnosis and understanding the behavior of this rare tumor.

## Methods

The Surgical Pathology database of the Department of Pathology and Laboratory Medicine at Aga Khan University Hospital (AKUH), was searched for cases reported as RH. Glass slides of all 3 cases were retrieved and reviewed, including the Hematoxylin & Eosin (H & E) stained slides as well as those of immunohistochemical (IHC) analysis. The clinical and follow-up data were obtained from medical records and by telephonic conversation. Radiological findings were also analyzed when available. 1 patient was lost to follow-up.

## Results

Three cases of RH have been reported from 1st June 2015 to 31st March 2020 at our department. Of these, 2 were females and 1 male. The age range was 18–50 years. Histologically, none of these cases showed features like nuclear atypia, spindle cell proliferation forming slit like spaces or more than rare mitotic figures that would suggest a diagnosis of angiosarcoma or Kaposi Sarcoma (KS).

### Case 1

**Fig. 1:**
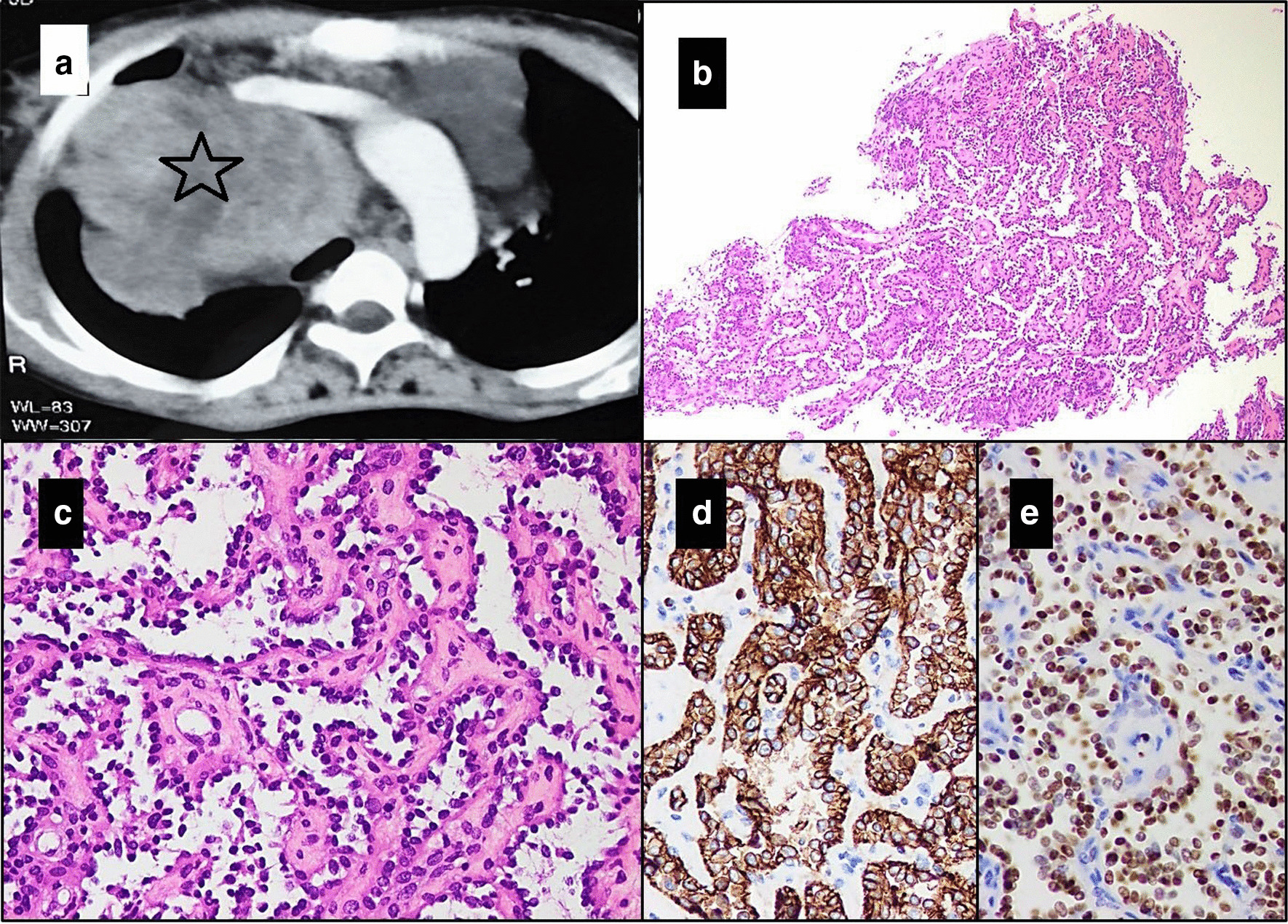
Case 1. **a** Computed tomography scan showing multiple heterogeneously enhancing soft tissue masses in the mediastinum, one in the right side is seen in the image (star). **b** Hematoxylin & Eosin stained sections of tumor showing characteristic arborizing retiform architecture of the vascular channels, appreciable on low power (20×). **c** The vascular channels are lined by endothelial cells with prominent hobnailing of the uniform appearing nuclei. These endothelial cells are positive for immunohistochemistry stains **d** CD31 and **e** ERG

A 50-year-old, non-smoker Pakistani female presented with shortness of breath and cough. She was known case of hypothyroidism and had previous history of pulmonary tuberculosis for which she had received anti-tuberculous therapy. She was evaluated for re-activation of pulmonary tuberculosis, but Gene Xpert on sputum turned out to be negative for Acid-fast bacilli. Her computed tomography (CT) scan showed multiple heterogeneously enhancing soft tissue masses in the mediastinum, the largest being 14.1 × 8.7 cm (Fig. 1a), which was compressing the pericardium. Encasement of the pulmonary trunk and superior vena cava by other masses was also observed. The inferior vena cava and heart chambers appeared dilated. Subsegmental collapse of the right middle and left inferior lobes of lung was also seen. Echocardiography showed ejection fraction of 40–45% with moderate mitral regurgitation and severe pulmonary hypertension. A suspicion of a lymphoporliferative disorder was raised on the basis of radiological findings. A core needle biopsy was received from one of the masses and it showed a tumor composed of interconnected arborizing vascular channels, lined by single layer of flattened cells with prominent hobnail nuclei. Significant nuclear atypia and mitotic figures were not seen. Mild lymphocytic infiltrate was also observed in the background stroma. The vascular nature of these channels was confirmed with positive staining for IHC stains of CD31, CD34 and ERG. Features of malignancy or any other type of vascular tumor were not present in this material. A diagnosis of RH was made (Fig. 1a–e). The patient is alive and healthy after 4 months of diagnosis. She has not taken any kind of treatment yet.

### Case 2

**Fig. 2:**
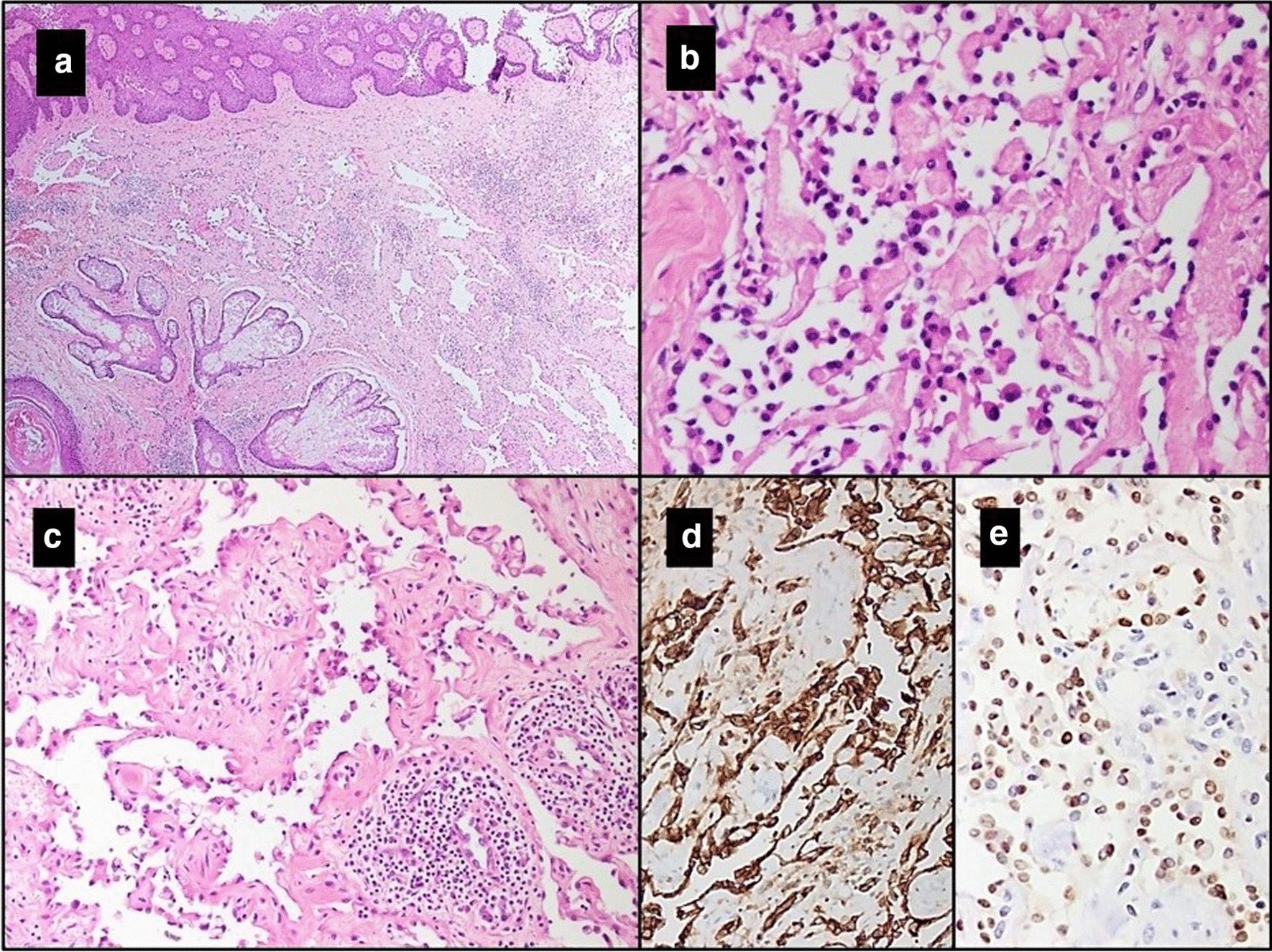
Case 2. **a** Hematoxylin & Eosin stained sections of tumor at scanning magnification (2×) within the dermis comprising of a network of anastomosing vascular spaces. **b**, **c** The vessels are lined by bland endothelial cells with hobnailing (40× & 20× respectively). **c** There are few lymphoid aggregates as well in the adjacent stroma. Positive expression for **d** CD31 and **e** ERG immunohistochemistry stains is observed

A 30-year-old Pakistani male presented with a lesion on the scalp at right temple. Radiological films or reports were not available for this case. An elliptical piece of skin was received, measuring 5 × 3 × 1 cm. It showed an ulcerated lesion, measuring 3 × 2.5 × 1 cm. This lesion had a hemorrhagic cut surface and was less than 1 mm away from the deep resection margin. Other margins were either 1 mm or more far from the lesion. Microscopic findings showed an angiomatous lesion in the dermis and deeper tissue, composed of dilated slit-like anastomosing vascular channels. These vascular channels were lined by endothelial cells with prominent hobnail nuclei, along with intracytoplasmic vacuole formation. The nuclei showed mild hyperchromasia and cytoplasm was eosinophilic. Scattered lymphoid aggregates were seen in the background. There was an absence of eosinophils within the lesion, which helped in differentiating it from angiolymphoid hyperplasia with eosinophilia, which is more common in the dermal tissue as compared to RH. IHC stains for CD31 and ERG were positive in these hobnail endothelial cells (Fig. 2a–e). The patient is alive and healthy at 7 months after the initial diagnosis. He has not received any radiation to the tumor site.

### Case 3

An 18-year-old Pakistani female presented with a hard growth on left 4th toe. Radiological films or reports were not available for this case. Amputated toe was received along with few separate skin covered tissue pieces. The amputated toe with intact nail and nail bed measured 5 × 2.5 × 2 cm. Separate tissue pieces measured 3 × 2 cm in aggregate. Cut surface showed a grey white, firm areas, measuring appro×imately 2 × 1 cm. These areas were present at 2.5 cm from the resection margin and were previously incised. Similar grey white areas were also noted in the separately present skin covered tissue pieces. The skin, soft tissue and bone at the resection margin appeared grossly uninvolved. Microscopically, the toe and separate pieces of skin showed a tumor infiltrating into the dermis and subcutis. Tumor was composed of narrow and anastomosing vascular channels, lined by hobnail endothelial cells. Focally these vessels were variably dilated and showed small intraluminal papillae with hyalinized fibrovascular cores. Tumor cells lacked nuclear atypia and increased mitotic activity. Mild lymphocytic infiltrate and focal hemosiderin pigment were also seen. Tumor cells demonstrated positive IHC expression for CD34, CD31 and ERG IHC stains. The underlying bone was not involved by the tumor. This patient was lost to follow up.

## Discussion

RH was first reported in 1994 by Calonje *et al.* who described it as a rare vascular tumor affecting patients over a wide age range without gender predilection [2]. The 5th edition of WHO classification of tumors of soft tissue and bone (2020) describes RH as a tumor affecting mostly younger population [1]. In contrast to WHO and similar to the earliest reported series, our cases showed a wide range of ages, from a teenager to a middle aged patient. Although, the most common site of involvement described in the literature is lower extremities [1, 2], several rare sites have also been reported, such as the pleura, mediastinum, head and neck region and spleen and so on [3, 5–7]. This diversity is apparent in our series with 2 of the cases arising at unusual paces that is the scalp and the mediastinum.

RH may present as multiple enhancing tumor nodules as reported in the earliest of descriptions of this entity [8], the same was observed in one of our patients, who presented with symptoms of cough and shortness of breath due to widespread mediastinal involvement. The other 2 cases had solitary tumors.

The cutaneous vascular tumors present mostly as indurated plaques [1], including hemangiomas, angiolymphoid hyperplasia with eosinophilia, kaposiform hemandioendothelioma, angiosarcoma and RH. One of our cases presented in a similar manner, with a lesion on the skin of scalp, while another one showed a variation on this theme. This latter patient had a hard growth on the toe, despite the tumor growing in the dermis and subcutis microscopically.

The first of our cases had an atypical presentation with shortness of breath. Such a presentation has only been described in the literature in a single case of RH involving the pleura [3]. The symptoms resulted from mass effect rather than direct involvement of the vital structures.

**Fig. 3:**
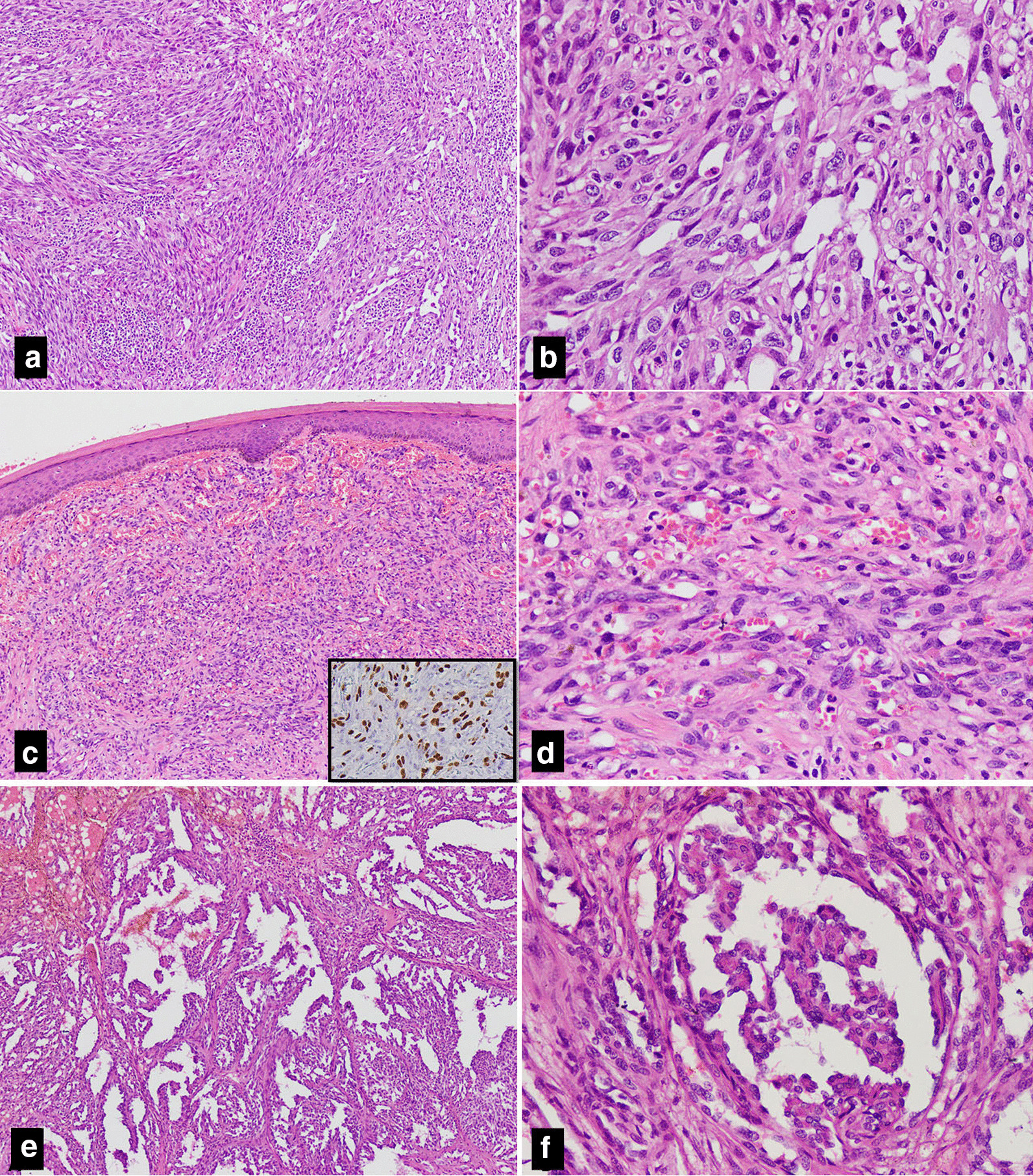
Angiosarcoma: **a** A cellular spindle cell tumor arranged in fascicles with vascular channels at periphery. **b** These cells show moderate cytologic atypia. Kaposi sarcoma: **c** Low power shows dermal proliferation of vessels and spindle cells. **d** Endothelial cells show mild atypia, cytoplasmic vacuoles and extravasated red blood cells. These cells are positive for ERG immunostain (inset). Papillary intralymphatic angioendothelioma (Dabska tumor): **e** Lesion composed of variable sized intraluminal proliferation of papillary tufts. **f** These tufts have hyaline cores. The lining cells have uniform round nuclei

RH can be commonly seen as a component of a complex vascular lesion, admixed with other components of intermediate category of vascular tumors. The most common of these being the papillary intralymphatic angioendothelioma (Dabska tumor). Composite hemangioendotheliomas, may also harbor areas morphologically indistinguishable from RH. These two entities belong to the most important differential diagnoses of RH and must be considered before making a final diagnosis of RH. Though Dabska tumor itself is an extremely rare entity, composite hemangioendotheliomas are slightly more common than RH and Dabska tumor as described in the WHO 2020 [1]. Histologically, Dabska tumor exhibits intravascular papillary proliferations lined by single layer of cells, with hobnailing of nuclei (Fig. 3e, f). The intraluminal papillae have hyalinized cores. Similar papillary structures may be seen in RH, as seen in 3rd case of our series. Anne-Fore Albertini et.al reported an unusual case of RH in a 6-year-old female, which showed areas of Dabska tumor. This tumor had developed at the site of a treated lymphangioma [9]. Some authors still believe these two entities to be closely related to each other and a definite distinction between the two may not be possible in all cases. However, presence of branching, interconnected vascular channels is a useful diagnostic clue and intraluminal proliferation with papillary architecture of Dabska tumor is completely absent in most cases of RH. Extravasation of red blood cells and lymphocytic infiltration in the background is more common in Dabska tumor. In addition, areas of lymphangioma at the periphery are seen in Dabska tumor [10]. IHC usually provides little help in distinction between these two tumors as both of these share positivity for vascular markers including CD31, CD34, ERG and Claudin-5. Podoplanin (D2-40), a marker of lymphatic lineage, is more frequently expressed in Dabska tumor than RH [1]. D2-40 was not performed on any of the cases described in this series.

It is also important to differentiate RH from more aggressive vascular tumors such as angiosarcoma, which may also show areas with hobnail endothelial cells, particularly in a small biopsy specimen (Fig. 3a, b). By definition, angiosarcoma shows nuclear atypia and mitoses in the endothelial cells, which is not seen in RH. KS should also be considered in the differential diagnosis of RH as both of these tumors occur in the dermis and subcutaneous tissue.

KS is usually considered in a patient who is immunocompromised, but this piece of information may not be available in every case. Characteristic histological features of KS include atypical spindle cell proliferation with intervening slit-like vascular spaces (Fig. 3c, d). Few cases of KS may show hobnail nuclei and retiform pattern of vascular channels. However, neither spindle cell proliferation nor slit-like spaces are observed in RH. IHC stain for Human Herpes Virus 8 (HHV8) may also be helpful in difficult cases as KS is positive and RH is negative for HHV8 immunostaining [11].

All three cases of this series showed characteristic morphological and IHC features of RH. Any other patterns to render a diagnosis of composite hemangioendothelioma were not seen, nor were there any features of malignancy.

RH demonstrates locally aggressive behavior as recurrence is reported in 60% cases. These recurrences are multiple and develop over many years. In addition, there is no evidence to suggest that deep seated tumors fare worse than their superficial counterparts. In contrast, Dabska tumor shows a lower comparative recurrence rate of about 40% [10]. Metastasis to lymph nodes is seen in very few cases and distant metastasis has been observed in a single case [5]. KS and angiosarcoma exhibit malignant potential. KS can metastasize to lymph nodes and distant organs, albeit to a lesser extent than a conventional angiosarcoma, which is notorious for its aggressive behavior and a dismal outcome in most cases, despite aggressive treatment.

KS is known to be associated with HHV8, which mostly affects individuals with a compromised immunity, specifically patients with low T-cell counts. The virus promotes neo-angiogenesis along with tampering with the DNA of the infected cell. The result is an uncontrolled proliferation of phenotypically as well as genotypically modified endothelial cells [12].

In contrast, angiosarcomas exhibit epigenetic and oncogenic mutation patterns along with UV damage signatures, as described in a recent study [13]. The availability of more advanced molecular techniques has enabled researchers to find specific genetic mutations that can serves as targets for therapies, as are commonly available for some of the epithelial tumors. The author came across a recent paper, in which the role of Endoglin (CD105) mutations has been studied in angiosarcomas [14]. Endoglin has been found to act as a coreceptor for TGF-Beta signaling pathway and has been found on malignant endothelial cells of angiosarcoma. Manipulation of this particular gene may act as a novel therapeutic target for angiosarcomas. Although, its role in other vascular tumors has not yet been described.

The molecular defects seen in some cases of RH include rearrangements and fusions of YAP1 and MAML2 genes, also seen in cases of composite hemangioendotheliomas. This finding supports the debate about these two entities representing a morphological spectrum in a single tumor type [15].

RH is traditionally treated by complete surgical resection with negative margins. In cases of cutaneous involvement, this may be achieved by Mohs micrographic surgery [16]. For unresectable tumors and tumors with multifocal presentation, some chemotherapeutic agents have been described in the literature, like low doses of Cisplatin along with radiation therapy [17].

## Conclusion

RH is a rare tumor of intermediate biological behavior. It should always be considered in the differential diagnoses while evaluating small biopsies showing vascular proliferation in dermis, particularly those with a hobnail pattern of endothelial cells. It may occur as a component of a composite hemangioendothelioma, which makes it necessary to examine different areas of a large resection specimen with care, the latter being more important in order to completely rule out a possibility of an angiosarcoma or a KS, which may harbor areas morphologically similar to RH.

## Data Availability

Data and materials of this work are available from the corresponding author on reasonable request.

## Abbreviations

RH: Retiform Hemangioendothelioma
KS: Kaposi Sarcoma
IHC: Immunohistochemistry
HHV8: Human Herpes Virus 8
AKUH: Aga Khan University Hospital
CT: Computed Tomography
WHO: World Health Organization

## References

[1] Fletcher CDM, Bridge JA, Hogendoorn PCW, Mertens F. WHO classification of tumors of soft tissue and bone. 5th ed. Lyon: IARC; 2020.

[2] Calonje E, Fletcher CD, Wilson-Jones E, Rosai J. Retiform hemangioendothelioma. A distinctive form of low-grade angiosarcoma delineated in series of 15 cases. Am J Surg Pathol. 1994;18:115–25. 8291650

[3] Liu Q, Ouyang R, Chen P, et al. A case report of retiform hemangioendothelioma as pleural nodules with literature review. Diagn Pathol. 2015;10:194. 10.1186/s13000-015-0433-7 PMC4624164 26503428

[4] Bhutoria B, Konar A, Chakrabarti S, Das S. Retiform hemangioendothelioma with lymph node metastasis: a rare entity. Indian J Dermatol Venereol Leprol. 2009;75:60–2. 10.4103/0378-6323.45223 19172034

[5] Chen CW, Hsiao KH, Lu TJ, Lai CW. Retiform and epithelioid hemangioendothelioma arising from the spleen. Formos J Surg. 2019;52:147–50.

[6] Cakir E, Demirag F, Gulhan E, Oz G, Tastepe I. Mediastinal composite hemangioendothelioma. A rare tumor at an unusual location. Tumori J. 2009;95(1):98–100. 10.1177/030089160909500117 19366065

[7] Mondal A, Das M, Chatterjee U, Datta C. Retiform hemangioendothelioma: an uncommon vascular neoplasm. Indian J Pathol Microbiol. 2020;63(1):122. 10.4103/IJPM.IJPM_237_19 32031140

[8] Duke D, Dvorak A, Harris TJ, Cohen LM. Multiple retiform hemangioendotheliomas. A low-grade angiosarcoma. Am J Dermatopathol. 1996;18:606–10. 10.1097/00000372-199612000-00010 8989934

[9] Albertini AF, Brousse N, Bodemer C, Calonje E, Fraitag S. Retiform hemangioendothelioma developed on the site of an earlier cystic lymphangioma in a six-year-old girl. Am J Dermatopathol. 2011;33(7):e84–7. 10.1097/DAD.0b013e31821b0a9f 21915027

[10] Goldblum JR, Weiss SW, Folpe AL. Enzinger and Weiss’s soft tissue tumors. London: Elsevier Health Sciences; 2020.

[11] DePond W, Said JW, Tasaka T, de Vos S, Kahn D, Cesarman E, Knowles DM, Koeffler HP. Kaposi’s sarcoma-associated herpesvirus and human herpesvirus 8 (KSHV/HHV8)-associated lymphoma of the bowel: report of two cases in HIV-positive men with secondary effusion lymphomas. Am J Surg Pathol. 1997;21(6):719–24. 10.1097/00000478-199706000-00013 9199651

[12] Dupin N. Update on oncogenesis and therapy for *Kaposi sarcoma*. Curr Opin Oncol. 2020;32(2):122–8. 10.1097/CCO.0000000000000601 31815777

[13] Chan JY, Lim JQ, Yeong J, Ravi V, Guan P, Boot A, Tay TK, Selvarajan S, Nasir ND, Loh JH, Ong CK. Multiomic analysis and immunoprofiling reveal distinct subtypes of human angiosarcoma. J Clin Invest. 2020;130(11):5833–46. 10.1172/JCI139080 PMC7598061 33016928

[14] Sakamoto R, Kajihara I, Miyauchi H, Maeda-Otsuka S, Yamada-Kanazawa S, Sawamura S, Kanemaru H, Makino K, Aoi J, Makino T, Fukushima S. Inhibition of endoglin exerts anti-tumor effects through the regulation of non-Smad TGF-β signaling in angiosarcoma. J Invest Dermatol. 2020. 10.1016/j.jid.2020.01.031. 32142796

[15] Antonescu CR, Dickson BC, Sung YS, et al. Recurrent YAP1 and MAML2 gene rearrangements in retiform and composite hemangioendothelioma. Am J Surg Pathol. 2020;44(12):1677–84. 10.1097/PAS.0000000000001575 PMC7773139 32991341

[16] Keiler SA, Honda K, Bordeaux JS. Retiform hemangioendothelioma treated with Mohs micrographic surgery. J Am Acad Dermatol. 2011;65(1):233–5. 10.1016/j.jaad.2009.12.014 21679834

[17] Hirsh AZ, Yan W, Wei L, Wernicke AG, Parashar B. Unresectable retiform hemangioendothelioma treated with external beam radiation therapy and chemotherapy: a case report and review of the literature. Sarcoma. 2010;26:2010. 10.1155/2010/756246 PMC2948930 20936126

